# Targeted degradation of a‐synuclein via the AdPROM system prevents PFF‐induced a‐synuclein aggregation

**DOI:** 10.1002/alz.088682

**Published:** 2025-01-09

**Authors:** Bill Carton, Géraldine Gelders, Nur Kocaturk, Sascha Roth, Thomas Macartney, Joke Van Elsen, Louis De Muynck, Arjan Buist, Diederik Moechars, Gopal Sapkota

**Affiliations:** ^1^ MRC Protein Phosphorylation and Ubiquitylation Unit, Dundee, Scotland United Kingdom; ^2^ Johnson & Johnson Innovative Medicine, Beerse Belgium

## Abstract

**Background:**

Accumulation of misfolded a‐synuclein protein in intracellular inclusion bodies of dopaminergic neurons underlies the pathogenesis of synucleinopathies, which include Parkinson’s Disease (PD), Dementia with Lewy Bodies (DLB) and Multiple System Atrophy (MSA). Therefore, clearance of misfolded α‐synuclein from dopaminergic neurons could in principle offer a an approach for modifying synucleinopathies, which currently remain untreatable.

**Method:**

In this study, we employ the Affinity‐directed PROtein Missile (AdPROM) system consisting of the substrate receptor of the CUL2‐E3 ligase complex VHL and a nanobody selectively recognising the human α‐synuclein protein

**Result:**

We demonstrate targeted degradation of endogenous α‐synuclein from human cell lines with exquisite selectivity. We further demonstrate that targeted degradation of α‐synuclein prevents the pre‐formed fibril‐ (PFF‐) induced aggregation of α‐synuclein in primary neurons derived from rats expressing human α‐synuclein. We will report at the meeting our progress on ongoing *in vivo* studies in a mouse model of α‐synucleinopathy

**Conclusion:**

Our findings demonstrate that targeted degradation of α‐synuclein in vitro and in vivo with AdProms is feasible.

